# Designing a wholly online, multidisciplinary Master of Cancer Sciences degree

**DOI:** 10.1186/s12909-023-04537-1

**Published:** 2023-07-31

**Authors:** Julia Lai-Kwon, Sathana Dushyanthen, David Seignior, Michelle Barrett, Femke Buisman-Pijlman, Andrew Buntine, Robyn Woodward-Kron, Grant McArthur, David L Kok

**Affiliations:** 1 Victorian Comprehensive Cancer Centre (VCCC) Alliance, Melbourne, Australia; 2 Department of Medical Education, Melbourne Medical School, Faculty of Medicine, Dentistry and Health Sciences, University of Melbourne, Melbourne, Australia; 3 Peter MacCallum Cancer Centre, 305 Grattan St, Melbourne, 3000 Australia; 4 Centre for Digital Transformation of Health, Faculty of Medicine, Dentistry and Health Sciences, University of Melbourne, Melbourne, Australia; 5 Melbourne School of Professional and Continuing Education, University of Melbourne, Melbourne, Australia; 6 Department of Clinical Pathology, Faculty of Medicine, Dentistry and Health Sciences, University of Melbourne, Parkville, VIC Australia

**Keywords:** Oncology, Medical education, Postgraduate education, Online education, cancer education, Healthcare professional education, Interprofessional learning, Workforce, codesign, Consumer informed, Professional development

## Abstract

**Background:**

Improving oncology-specific knowledge and skills of healthcare professionals is critical for improving the outcomes of people with cancer. Many current postgraduate education offerings may be inaccessible to busy professionals, contain minimal consumer input or do not focus on the multidisciplinary nature of cancer care. In response to these needs, a Master of Cancer Sciences degree was developed. Our aim is to describe the development of the Master of Cancer Sciences.

**Methods:**

We describe the development of the Master of Cancer Sciences, including its theoretical and its pedagogical underpinnings.

**Results:**

Our approach to curriculum design was guided by Kern’s Six-Step Approach to Medical Curriculum and underpinned by the Seven Principles of Online Learning. These approaches were further underpinned by the Cognitive Theory of Multimedia Learning which informed our approach to audio and visual information design. The pedagogy is interactive, experiential, interprofessional and importantly, includes consumers as educators. In practice, learning activities include peer feedback, multidisciplinary team meeting simulations, group work and clinical role plays. The online environment was visually shaped through infographics, high-quality educational videos and gamification.

**Conclusion:**

We have designed a Master of Cancer Sciences that is one of the first wholly online, cancer-specific Masters’ programs. Its industry-led curriculum using evidence-based pedagogical choices utilises a range of novel digital formats and integrates the consumer perspective to provide a holistic overview of the field. Quantitative and qualitative evaluation of learning outcomes is ongoing.

## Background

Improving the oncology-specific knowledge and skills of healthcare professionals is critical to improving the clinical outcomes of people with cancer. Knowledge generation in the cancer sphere has been rapid, but the lead-time between discovery and adoption into routine clinical practice remains long, leading to inequities in care [1]. High quality, interdisciplinary, accessible oncology education programs can increase the pace of dissemination and adoption of research findings which in turn can lead to improved patient outcomes [2].

Traditional postgraduate teaching programs have numerous limitations that limit their appeal, feasibility and applicability to oncology professionals. In particular, most oncology education offerings have typically been delivered in blended format requiring at least some on-campus attendance [3–5]. The requirement for on-campus attendance at fixed times significantly reduces accessibility for both busy professionals and those living in rural or remote locations, and other locations interstate or internationally. This hurdle, compounded by the underlying variability in motivation and time which healthcare professionals can dedicate to acquiring new knowledge [6] has contributed to a very low proportion of working oncology professionals pursuing advanced postgraduate degrees. Additionally, real-world applicability of oncology educational offerings has not always been in line with the latest advances in clinical practice. In particular, integration of the patient perspective is often limited to simulated patients in roleplay scenarios, rather than exploring the full spectrum of ‘consumer-informed’ educational practice. Furthermore, it has become increasingly clear that multidisciplinary care is a vital component in optimal cancer care [7, 8] but many courses focus just on specific craft groups such as medical oncologists or nurses and are thus not multidisciplinary in nature and have not applied interdisciplinary learning techniques as a core part of their curriculum.

Meanwhile, the educational sphere has had a major re-alignment in the 21st century, with an increasing focus on digital learning. Two factors may have contributed to this. Firstly, attitudes towards online learning have shifted substantially [9] and this has been further accelerated by the COVID-19 pandemic [10]. While there were some notable cases of oncological educational programming moving online prior to this, they were in the minority until 2020 [11, 12]. Secondly, there has been significant evolution in the digital learning methods deployed with sophisticated, engaging, pedagogically-driven solutions now available. Several studies have demonstrated that learning outcomes from online courses both generally, and in health education, are equivalent, if not superior to, those delivered in person [13–15]. The convenience and flexibility of digital learning, as well as the rapid translation of new knowledge ensuring currency, means it is particularly well-suited to the oncology field [16].

In response to this gap in educational offerings and driven by a desire to grow the breadth and depth of the cancer workforce, the Victorian Comprehensive Cancer Centre (VCCC) Alliance partnered with the University of Melbourne to produce a bespoke new degree, the Master of Cancer Science. To the best of our knowledge at the time of the degree’s inception and still to this day, there are no other Masters’ degrees offered worldwide that are oncology-focussed, specifically emphasise interdisciplinary practice, are centred around the patient experience and are delivered fully online [17].

The aim of this paper is to describe the theoretical and pedagogical underpinnings, development, implementation, and preliminary evaluation of the Master of Cancer Sciences.

### Program description

The Master of Cancer Sciences is the first cancer-specific, multidisciplinary, and wholly online Masters program in Australia. Developed and delivered in conjunction with the VCCC Alliance (a partnership of 10 research, academic and clinical institutions in Victoria, Australia) and the University of Melbourne, its primary goal is to build future oncology workforce capacity in Australia and internationally. Graduates are equipped with the specialist knowledge needed to work in clinical care and cancer research, which can improve the experiences and outcomes for patients and their families. Participants gain a comprehensive understanding of cancer biology, research, and clinical care; a detailed knowledge of the historical, societal and political context of cancer care; and the ability to design and conduct a substantial research project in an ethical manner.

The course is targeted at scientists and clinicians from a range of backgrounds, including medical practitioners, nurses, allied health professionals, pharmacists, research scientists, clinical trials professionals, and industry and pharmaceutical professionals. Entry requirements for the degree were completion of an undergraduate degree with honours in an appropriate discipline or at least two years of relevant work experience; or at least eight years of relevant work experience with written and analytical skills suitable for postgraduate study. There were no limits to the number of students enrolled in the course.

**Fig. 1:**
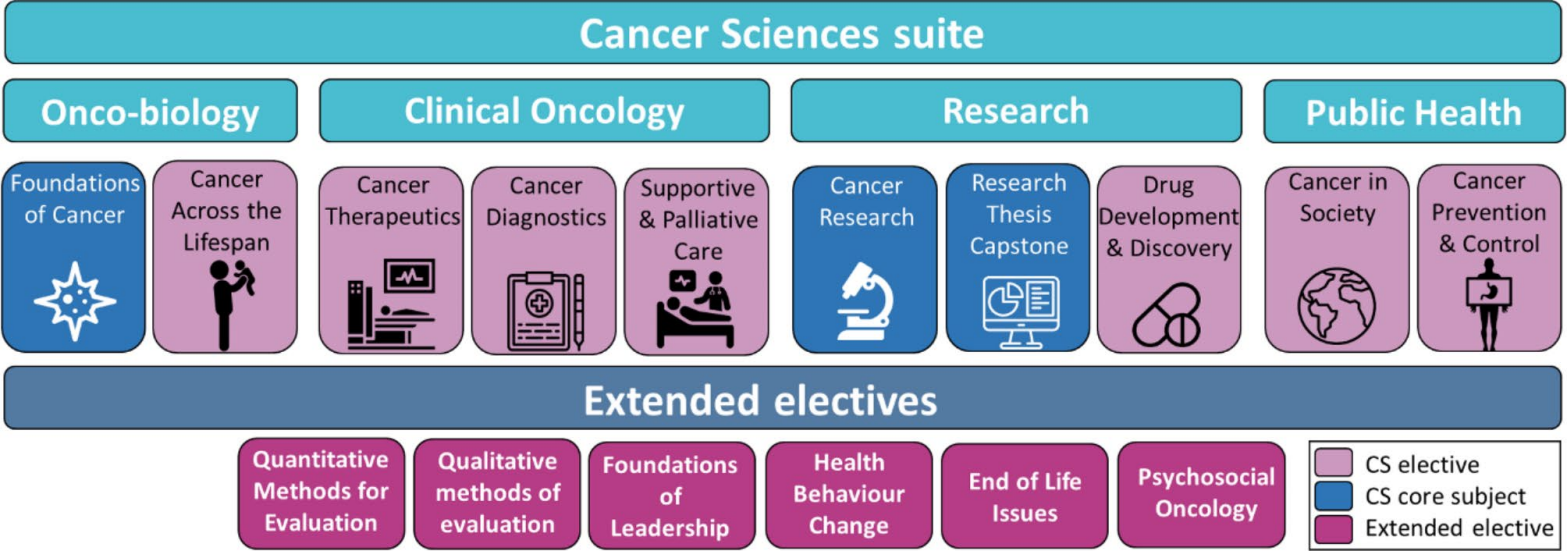
The Master of Cancer Sciences program has ten cancer-specific subjects that provide a holistic overview of the field and a suite of ‘extended’ electives to maximise relevance to the diverse backgrounds of potential students, cover the broad scope across the oncology sector and provide stimulating learning opportunities. CS: Cancer sciences

**Fig. 2:**
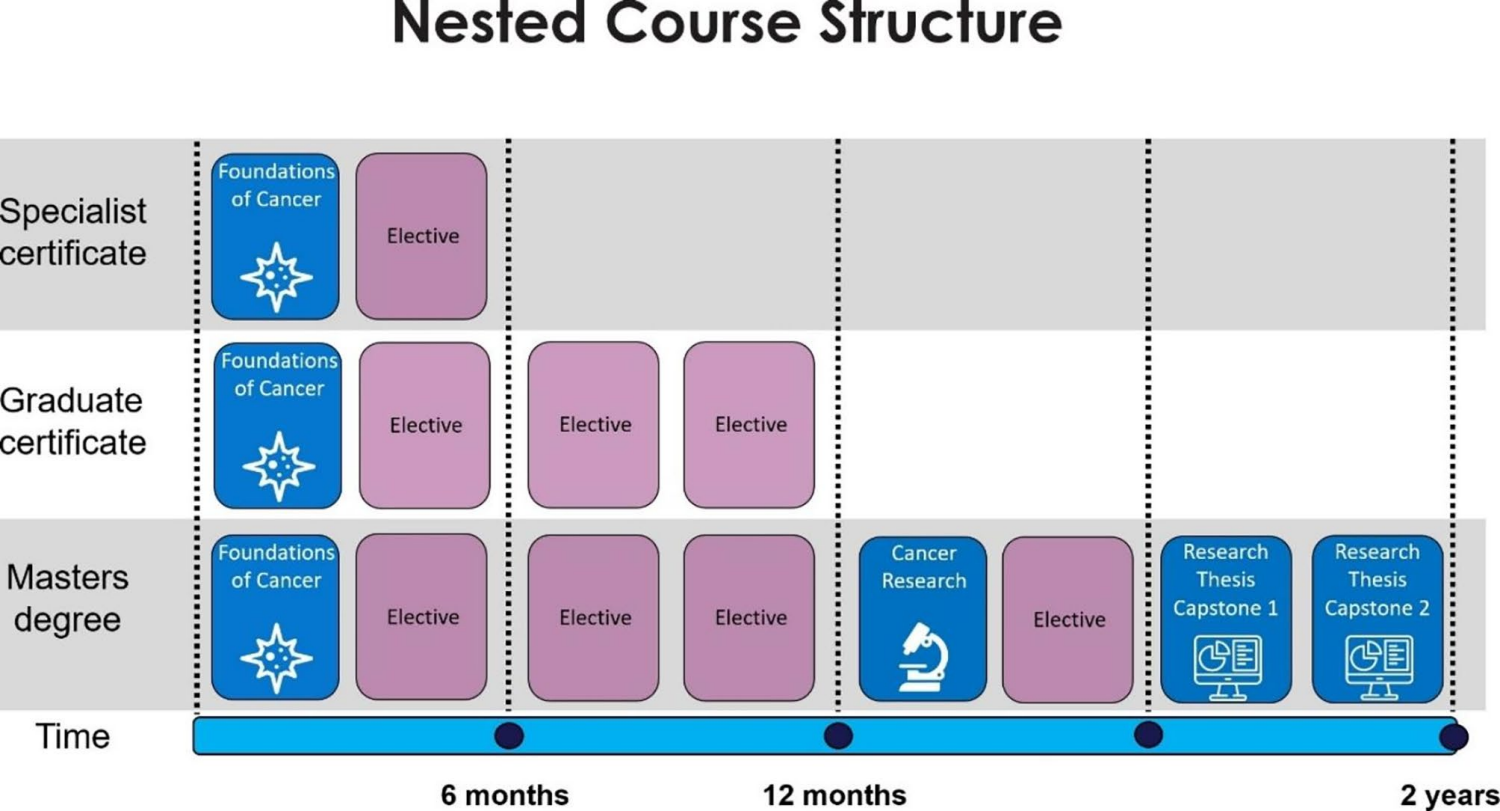
Nested course structure of the Master of Cancer Sciences

The course consists of four core subjects and thirteen electives, enabling students to customise the program to suit their individual interests and needs (Fig. 1). It is available as individual professional development subjects, cross-course electives, as a Specialist Certificate, Graduate Certificate or Masters depending on how much of the program is completed by the student (Fig. 2). Students are required to complete four core subjects and four electives to obtain the Masters degree. A six-month research capstone involving a systematic literature review, oral presentation and 4000-word monograph at the conclusion of the Masters provides a potential pathway to a PhD.

The wholly online curriculum consists of ten cancer-specific subjects that provide a holistic overview of the field and a suite of extended electives. Each subject was informed by a working party of approximately 30 subject matter experts (recruited through an expression of interest and direct invitation process) led by the subject coordinator (recruited through an expression of interest process). More than 270 subject experts from across the VCCC Alliance members and international experts from University of Oxford (UK), Columbia University (USA) and the Boehringer-Ingelheim Regional Centre (Austria) contributed to the program’s development and delivery. Subject coordinators, the working party and project team workshopped the subject’s curriculum outline of three lessons per week for eight weeks and then assigned the topics to experts in the field. Expert content was received in a templated format and then the learning design team suggested interactive and engaging modalities to translate the content. The industry-based subject matter expert-led approach of the Master of Cancer Sciences was distinct from the usual academic-led approach of the University of Melbourne. Consumers were engaged in the development and delivery of all subjects at multiple levels to ensure a focus on patient-centred care and research, including provision of advice on working groups, hosting journal clubs, providing consumer perspectives through interviews and panel discussions, and providing expert advice as Research Capstone supervisors.

### Program development

#### Theoretical and pedagogical underpinnings

The program was designed by content experts in collaboration with online learning designers and a team of experts with skills ranging from graphic design, video production to software programming at the University of Melbourne. Given the course is entirely delivered online, a careful theoretical and pedagogical approach was taken to provide the best possible learning and teaching experiences for students and teachers within the constraints and opportunities of online education. The overall design of the curriculum was guided by the Six-Step Approach to Medical Curriculum by Kern et al. (Table 1) [18] as well as the Seven Principles of Online Learning (Table 2) [19].

**Table 1:** Kern’s Six-Step Approach to Medical Curriculum

Steps	Approach in the Masters of Cancer Sciences
1. *Problem identification and general needs assessment*	• Stakeholder engagement e.g. within the VCCC Alliance, University of Melbourne • Board-level advice on educational landscape and need for Masters course
2. *Targeted needs assessment*	• Market analysis to identify needs within the Australian and international oncology workforce by an external consulting firm
3. *Goals and objectives*	• Identification of subject, course and aggregate course-level learning outcomes
4. *Educational strategies*	• Central coordinating team of educational and academic experts to ensure a cohesive curriculum and consistency of vision • Formation of multidisciplinary expert working parties for each of the ten cancer-specific subjects • Mapping of learning outcomes to course content and assessment tasks • Creation of course content using the Seven Principles of Online Learning (Table 2) and relevant pedagogical theories (Table 3)
5. *Implementation*	• Course delivered in 2019 • Multi-tiered promotional campaign Internal: University of Melbourne & the 10 VCCC Alliance Organisations External: Web (with SEO), social media, conferences, traditional media
6. *Evaluation*	• Formation of a preliminary evaluation framework • Quantitative evaluation methods: o University of Melbourne Student experience surveys conducted every 6 months during course participation o Customised Master of Cancer Sciences survey conducted 1-year post-graduation • Qualitative evaluation methods: o Qualitative study to assess impact on career trajectory and professional practice (in progress) • Planned refresh of course content to incorporating student feedback and ongoing developments in the cancer sphere (each subject has a yearly minor refresh and triennial major refresh)

**Table 2:** Seven Principles of Online Learning

Principle	Course examples
1. *Deep engagement and learning*	• Digital learning methods (infographics, educational videos, gamification) • Evidence-based case studies to show students where/how they can apply learnings within their own practice
2. *Interaction and feedback*	• Online interaction opportunities through discussion boards, group work • Regular interaction and live webinars with subject tutors • Course assessments with feedback • Peer review of assessments
3. *Flexibility and choice*	• Wholly online course accessible from any device anywhere in the world • Flexible format with core subjects and electives • Nested award format • Balance of synchronous and asynchronous learning activities
4. *Connection with world experts*	• Development and delivery of the course by ~ 300 academic, industry and consumer experts across the VCCC Alliance members, international experts from Oxford University (UK), Columbia University (USA) and Boehringer-Ingelheim Regional Centre (Austria) • Expert interviews and lectures • Interaction with academic and industry experts as research capstone supervisors
5. *Creation of a virtual scholarly community*	• Entry requirements to ensure students brought relevant workplace experience to the course • Video introductions to all course participants • Peer interactions through discussion boards, peer review of assessments, webinars

Given cancer professionals were likely to require flexibility to learn whilst maintaining active work in the sector, the Masters was designed to be a high quality, accessible, flexible program with immediately actionable clinical learnings and opportunities for reflective practice.

The creation of an online scholarly environment was of particular importance to ensure a positive student cohort experience, a sense of belonging, and to minimise isolation which may lead to disengagement in online learning. To do this, the Master of Cancer Sciences drew on elements of the Community of Inquiry (COI) model [20] which encourages focus on cognitive presence, social presence, and teacher presence for optimal student experience. The Masters balanced self-directed asynchronous student, peer, and teacher engagement through activities such as discussions and assessment tasks with synchronous engagement in the form of optional fortnightly webinars. This provided students with flexible and independent learning, supported by regular peer-to-peer and teacher ‘live’ touchpoints.

Key pedagogical theories informing the development of the Masters are shown in Table 3. One of the foremost of these was that of interprofessionality and interprofessional education. Interprofessional education is defined as when two or more professionals learn with, from and about each other to improve collaboration [21]. Given cancer is managed in multidisciplinary teams, this requires strong interprofessional, collaborative and team-based approaches. In the Masters, interprofessional learning was facilitated through collaborative pedagogical approaches such as peer feedback on oral and written presentations, discussion threads on clinical decision making, Multidisciplinary Team Meeting (MDT) simulations, group work and clinical role plays in synchronous sessions. These were based on the six domains of interprofessional education assessment [22]- role understanding, interprofessional communication, interprofessional values, coordination and collaborative decision making, reflexivity and teamwork.

**Table 3:** Pedagogical strategies to address the specific needs of cancer professionals

Key issue	Pedagogy	Solution	Course examples
Diverse geographic location of cohort	• Online delivery method	• Wholly online course, accessible from any device and designed to be responsive.	-
Heterogeneity of cohort in terms of discipline and level of experience	• Flexibility of curriculum design • High touch supervision	• Flexible format with core subjects and electives • Nested award format • Low bar to entry with a stepwise approach to resource levelling • Regular interaction and live webinars with subject tutors • Practical toolkits	-
Time poor participants with competing priorities and distractions	• Cognitive load theory • Cognitive Theory of Multimedia Learning • Visual Information Design Theory	• Present material in succinct, ‘chunked’ packages • Infographics • Screen-based learning methods • Interactivity and gamification • Augmented/ visual reality	• Screen based learning methods examples: https://my.visme.co/view/x4evv81w-mcs-catl-pregnancy-physiology https://my.visme.co/projects/pvgr4w1g-5a-communication-about-informational-and-emotional-concerns#s1 • Augmented/ visual reality example: https://www.youtube.com/watch?v=3NXVyaNkElU • https://www.youtube.com/watch?v=Ov8N2BcluO4
Complexity of oncological material	• Cognitive load theory • Cognitive Theory of Multimedia Learning	• Tailored choice of delivery medium to suit individual lesson • Infographics • Bespoke educational videos allowing dynamic on-screen drawing, eye contact, purposeful visual cuing • Branching scenarios to promote experiential learning • Gamification to allow practice, reinforcement, and review of key concepts	• Bespoke educational videos examples: https://www.youtube.com/watch?v=xHlNXzfCv0o https://www.youtube.com/watch?v=bGBtG_OsjeI • Branching scenarios example: https://youtu.be/w_WcvaecYKg
Need for knowledge and skills applicable to practice	• Applied learning design • Industry-based and sector-leading teaching team • Reproduction of real tasks as assessment tasks	• Patient and multidisciplinary-focused case studies • Expert interviews • Panel discussions • Mock multidisciplinary meetings to replicate real-world interactions • Assessments promoting application of learnings to the real-world context	• Patient story example: https://www.youtube.com/watch?v=zU9OHFO4Yks • Panel discussion: https://www.youtube.com/watch?v=UJVGruh8s3o
Need to promote multidisciplinary learning	• All students come with prior knowledge and experience allowing them to contribute meaningfully to the course • Interprofessionality and interprofessional education	• Discussion boards to facilitate learning from peers • Group work • Clinical role plays	-
Integration of the lived experience of consumers throughout the curriculum design and delivery	• Consumers as educators • Simulation • Co-design • Integrating lived experience in research	• Expert consumer interviews and reflections • Consumers as expert panellists • Simulated clinician / patient scenarios • Consumers as research capstone supervisors to ensure consumer inclusion in research design	• Consumer interviews example: https://youtu.be/SK-AMigWX4E

Several other key educational theories underpinned the Masters to address the specific educational needs of learners in the cancer domain. Cognitive Load Theory, which divides memory into sensory, working, and long-term memory, indicates that working memory can easily be overloaded, resulting in a limited ability to retain information [23]. Therefore, we focused on providing information in succinct packages, which were revisited during the learning task to enhance linkages to long-term memory and paced learning activities with appropriate intermissions to reset working memory. Visual Information Design theory was also critical, recognising that conveying information visually is a highly effective teaching method [24]. Good visual representations can reduce a learner’s extraneous computational load, increasing capacity for more important learning processes such as long-term memory encoding and accurate representation of complex ideas.

Patients as educators [25] and integrating the patient experience in research design [26] were two very important philosophies that informed the pedagogy and solutions applied. Every subject’s expert reference group had at least one person with a lived experience of cancer involved in designing the curriculum, producing and reviewing content. Each subject also interviewed one, or in several cases, more than one patient as part of the course content. Patients were also invited as webinar guests to provide a direct perspective on webinar presentations.

#### Digital learning methods

Given that the Masters is wholly online, the Cognitive Theory of Multimedia Learning was critical in informing the screen-based learning methods employed in the course [27–29]. This theory describes how the brain interprets multimedia presentations, selecting words, pictures and auditory information and dynamically organising these to produce logical mental constructs. Therefore, design principles focusing on the provision of coherent visual and verbal information can help learners select the relevant words and images and thus reduce the load on either the auditory or visual processing channels.

Screen-based learning methods are the most common method of digital delivery and provide an excellent learning experience with clear qualitative and pedagogical benefits over traditional teaching methods if selected and designed appropriately.

**Fig. 3:**
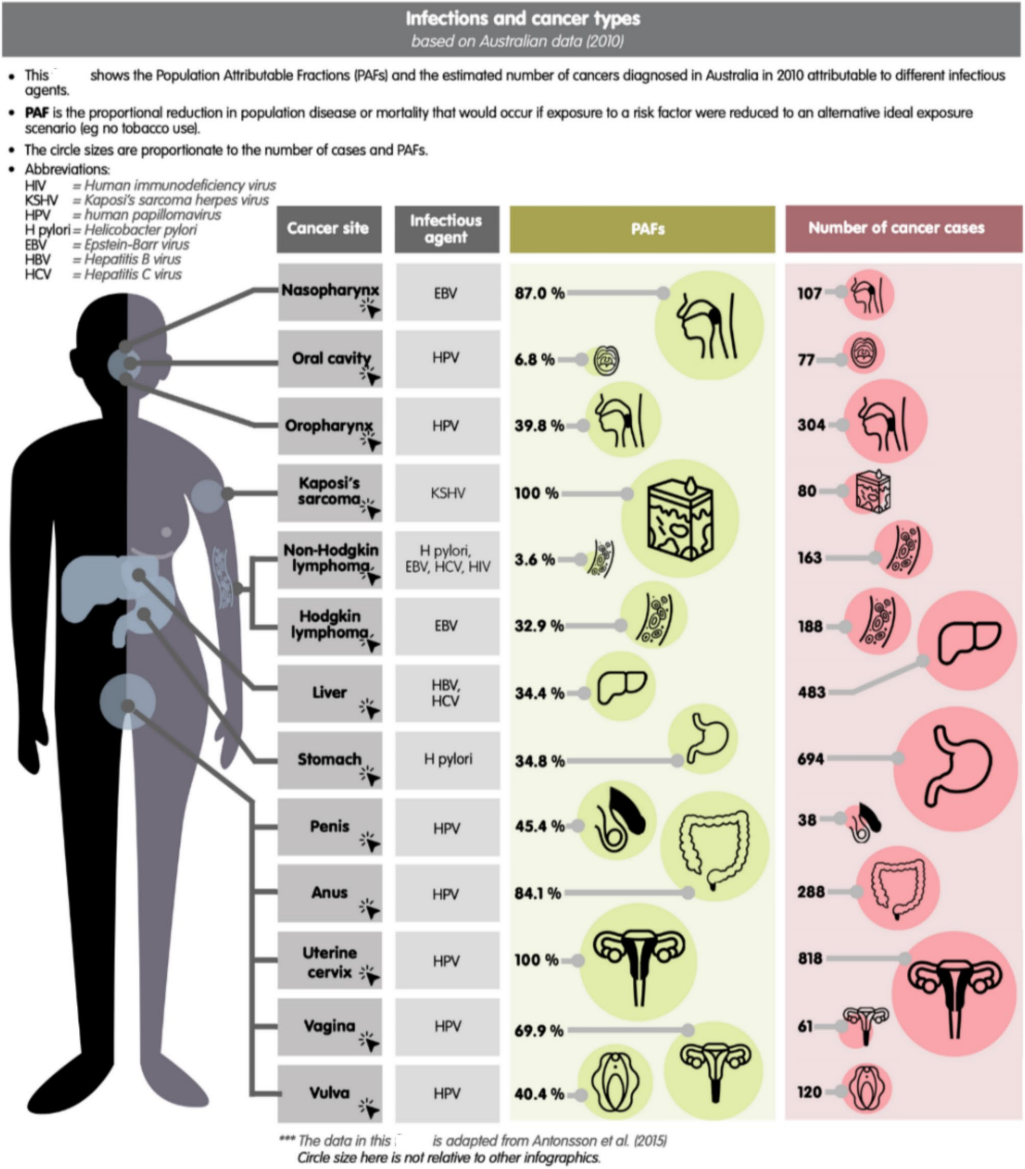
An interactive infographic from the Master of Cancer Science [31, 32]. See link in reference for original interactive version

*Infographics/ visual graphics* are visual representations of information combining data, charts, icons and illustrations with relatively minimal text [30]. We utilized infographics to present material about complex oncology processes and pathways succinctly and to enable students to engage, process and retain information better than text alone [24]. An example of an infographic from the Masters is shown in Fig. 3.

*Educational videos*, including documentary-style presentations, immersive 360-degree videos including procedural demonstrations, virtual reality, expert interviews, and panel discussions were also used throughout the Masters. Educational videos have developed beyond basic instructional videos or recorded lectures with an accompanying slide deck towards sophisticated videos drawing upon multimedia learning principles [33]. We utilized careful curation of both the verbal and non-verbal content to maximise cognitive processing, control over the duration of videos to regulate the speed at which the learner processes the information, and ‘weeding’ of all extraneous or redundant information to optimise mental capacity. Presenters were often shown to allow dynamic on-screen drawing, eye contact and purposeful visual cuing (drawing attention to relevant on-screen information) which have all been shown to enhance learning [34].

*Gamification elements* were also included throughout the course. We incorporated elements of gameplay into real-word activities and behaviours for the purpose of learning and is increasingly being used in a medical education context. Pedagogically, gamification enables active learning by practicing, reinforcing, reviewing and applying knowledge, troubleshooting and problem solving, which can enhance a learner’s metacognitive strategies and promote deep learning [35]. Several examples of gamification already exist in oncology education, such as the Cancer Research UK’s Citizen Science Project [36].

Other methods of content delivery included augmented/ virtual reality simulation; branching scenarios to promote experiential learning; contextualised, consumer and multidisciplinary-team focused, evidence-based case studies, expert interviews, and panel discussions; mock multidisciplinary team meetings; and live, interactive webinars and podcasts.

The course featured a program of assessments which drew upon student’s personal work experiences and promoted application of learnings to their real-world context. Assessment was enhanced through discussion forums and peer-review of assessments.

All students are also required to undertake a research project to obtain the Masters degree. This allowed them to learn and develop research skills to apply in clinical practice. This also facilitated a different level of student-teacher interaction as each student was allocated at least one research mentor/ supervisor from the internal University team in addition to their external research supervisor.

### Development of a preliminary evaluation framework

The preliminary evaluation framework for the Masters included administration of the online University of Melbourne Student Experience Survey every 6 months during course participation, and a customised in-house survey conducted 1 year after graduation regarding course satisfaction and self-perception of competence. Due to COVID-19-related university disruptions, the Student Experience Survey was not conducted during 2020 and 2021 and therefore data is not presented here.

We performed a detailed review of the demographics of the 122 students that enrolled in the first two delivery cohorts (2019 and 2020). Students were from a range of ages (21–30 years, n = 55, 45.08%), genders (female, n = 93, 76.23%), educational backgrounds (science, n = 33, 27.05%), occupations (pharmacy, n = 21, 17.21%) and geographical locations (metropolitan, n = 82, 67.21%), adding to the depth of interdisciplinary learning and the richness of multidisciplinary perspectives in the online community (Table 4).

**Table 4:** Demographic analysis of the 2019 & 2020 Master of Cancer Sciences cohort

Demographic	Categories	Number of participants	Percentage
Gender	Female	93	76.23%
	Male	28	22.95%
	Unknown	1	0.82%
Age range	21–30 years	55	45.08%
	31–40 years	30	24.59%
	41–50 years	24	19.67%
	50 + years	6	4.92%
	Unknown	7	5.74%
Location of participants	Australia- metropolitan	82	67.21%
	Australia- regional or rural	29	23.77%
	International	4	3.28%
	Unknown	7	5.74%
Educational background	Science	33	27.05%
	Medicine	29	23.77%
	Nursing	17	13.93%
	Pharmacy	20	16.39%
	Allied Health	7	5.74%
	Physiotherapy	6	4.92%
	Radiation therapy	1	0.82%
	Dentistry	1	0.82%
	Unknown	8	6.56%
Occupation	Pharmacy	21	17.21%
	Nursing	12	9.84%
	Clinical trials	12	9.84%
	Research	11	9.02%
	Medical- non oncology	11	9.02%
	Medical- oncology	7	5.74%
	Allied health	7	5.74%
	Physiotherapy	6	4.92%
	Other	6	4.92%
	Hospital administration	2	1.64%
	Dentistry	1	0.82%
	Unknown	26	21.31%

**Fig. 4:**
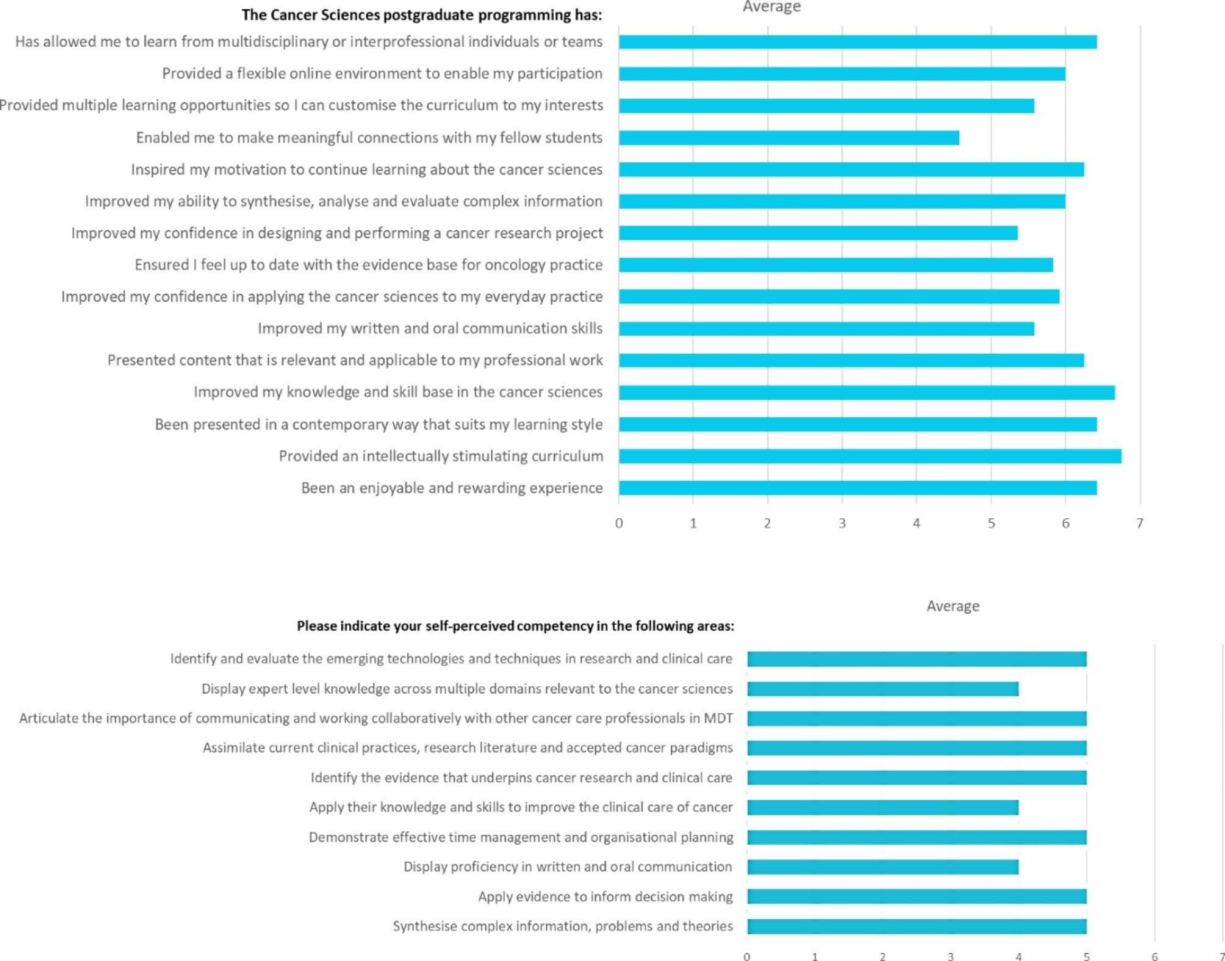
Results from a survey of students after 1 year of study in the Master of Cancer Science. Responses were across multiple domains and self-perceived competence based on graduate attributes and learning outcomes. All responses were rated out of 7

A 1-year follow up survey has been completed for the student cohort who commenced the Masters in January 2019. A series of questions aligned with the University’s graduate attributes and expected learning outcomes were surveyed after 1 year of study in the degree. Fifty-six students were surveyed (response rate: 61%). Average satisfaction with the program was 6/7 (87%) and average self-perception of competence was 5.9/7 (84%) after 1 year of student feedback (Fig. 4). A 5-year follow up survey will be conducted to track the career trajectory of students. Further course evaluation is ongoing and will be reported, including a qualitative study examining the impact of the course on career trajectory and professional practice and ongoing University and customised surveys as described above.

### Future plans

Given the rapid ongoing advancement of the cancer field, continuous refreshment of course content is imperative to ensure the most up-to-date content is taught. A review of the content of each subject is conducted preceding each delivery period to consider student and faculty feedback from the prior delivery and emergent research in the sector. In addition, a more major structural review and refresh of each subject is scheduled for every three years.

Having established a strong initial student base, attracting and retaining students from broad geographical locations with more rural, regional, interstate, and international students to increase educational research and professional development across the sector is also planned. Repurposing of course content for a range of other educational programs is also planned due to demand for course content in other areas, including for undergraduate and graduate medical degrees, Massive Open Online Courses, and micro-certificates for continuous professional development in cancer sciences.

## Conclusion

We have developed a Master of Cancer Sciences that is one of the world’s first wholly online, cancer-specific Masters’ programs. The course’s development has been informed by several key theoretical and pedagogical theories addressing interprofessionality, multidisciplinary learning, cognitive load and visual information design. It utilises a range of novel digital formats to address the evolving educational needs of healthcare professionals. Evaluation of the impact of the Masters on graduate career trajectory and impact on professional practice is ongoing.

## Data Availability

The datasets used and/or analysed during the current study available from the corresponding author on reasonable request.
